# Ecuadorian Cacao Mucilage as a Novel Culture Medium Ingredient: Unveiling Its Potential for Microbial Growth and Biotechnological Applications

**DOI:** 10.3390/foods14020261

**Published:** 2025-01-15

**Authors:** Tania María Guzmán-Armenteros, Luis Santiago Guerra, Jenny Ruales, Luis Ramos-Guerrero

**Affiliations:** 1 Departamento de Ciencia de Alimentos y Biotecnología (DECAB), Escuela Politécnica Nacional (EPN), Quito 170525, Ecuador or tamaguzm@espol.edu.ec (T.M.G.-A.); jenny.ruales@epn.edu.ec (J.R.); 2 Facultad de Ingeniería Mecánica y Ciencias de la Producción, Carrera de Ingeniería en Alimentos, Escuela Superior Politécnica del Litoral, Campus Gustavo Galindo, km 30.5 Vía Perimetral, Guayaquil 090902, Ecuador; 3 Carrera de Medicina, Facultad de Ciencias Médicas, Universidad Central del Ecuador (UCE), Campus El Dorado, Quito 170403, Ecuador; lsguerrap@uce.edu.ec; 4 Grupo de Investigación en Bio-Quimioinformática, Carrera de Ingeniería Agroindustrial, Facultad de Ingeniería y Ciencias Aplicadas, Universidad de Las Américas (UDLA), Quito 170503, Ecuador

**Keywords:** cacao mucilage, culture medium, bacteria, yeasts, fungi, mix design, microbial growth

## Abstract

Cacao mucilage is typically disposed of during processing, yet its abundant content of organic compounds, polysaccharides, and nutrients renders it valuable for various applications. This scientific study investigates the suitability of cacao mucilage as an alternative culture medium for *Lactiplantibacillus plantarum*, *Saccharomyces cerevisiae*, and *Aspergillus niger*, aiming to provide a viable alternative to traditional media. Through a mixed-design approach, the powdered mucilage, peptone, and yeast extract ingredients were optimized using the recovery rates of each micro-organism as the response variable. The optimal formulation of the medium, consisting of 49.6% mucilage, 30% yeast extract, and 20.9% peptone, resulted in remarkable microbial recovery rates. *L. plantarum* achieved an outstanding recovery rate of 98.18%, while *S. cerevisiae* and *A. niger* exhibited recovery rates of 90.57% and 89.90%, respectively. Notably, these recovery rates surpassed those obtained using conventional culture mediums. Thus, cacao mucilage emerges as an effective component for formulating a natural culture medium that facilitates the growth of yeasts, lactic acid bacteria, and fungi. The yeast extract peptone mucilage (YPM) medium demonstrated enhanced growth, particularly for yeasts and lactic acid bacteria, with recovery rates exceeding 90%. Conversely, *A. niger* displayed a relatively lower recovery rate. These findings emphasize the potential of cacao mucilage as a valuable resource for preparing natural culture media that promotes the growth of yeasts, lactic acid bacteria, and fungi, offering promising prospects for various applications in microbiology and biotechnology.

## 1. Introduction

Cacao mucilage, a nutrient-rich by-product of cacao production, has gained increasing attention for its potential applications across various industries, transforming what was once considered waste into a valuable resource. With its high sugar content, bioactive compounds, and dietary fiber, cacao mucilage offers promising uses in food production, biofuel generation, and nutraceutical development. For instance, it has been effectively used to produce fermented beverages with desirable physicochemical and sensory properties, offering a sustainable alternative to traditional waste disposal methods [1]. Additionally, its sugar-rich composition makes it a viable substrate for second-generation bioethanol production, helping to reduce environmental waste while contributing to renewable energy sources [2,3].

Beyond its role in beverage and biofuel production, cacao mucilage is rich in bioactive compounds like polyphenols and flavonoids, which exhibit strong antioxidant activities. These properties make it a valuable ingredient in the development of functional foods and nutraceuticals [4,5,6]. Moreover, lactic acid bacteria isolated from cacao mucilage have demonstrated biopreservative capabilities, inhibiting the growth of pathogens such as *Salmonella* and *Escherichia coli* in meat products, without affecting their physicochemical properties [7]. Its dietary fiber content, along with its ability to retain water and oil, further enhances its applicability in food enrichment and nutritional enhancement [5,6].

Three microbial species commonly isolated from cacao mucilage: *Saccharomyces cerevisiae*, *Lactiplantibacillus plantarum*, and *Aspergillus niger,* demonstrate remarkable potential in various industries. *S. cerevisiae*, a versatile yeast, is essential for alcohol production due to its ability to efficiently convert sugars into ethanol, contributing to both alcoholic beverage and biofuel industries [8,9]. Additionally, it holds promise in biotechnology for producing high-value compounds [10]. *L. plantarum*, a lactic acid bacterium, exhibits probiotic properties and plays a key role in vitamin production, making it valuable in food and nutraceutical applications [11,12,13]. *A*. *niger*, a filamentous fungus, excels in producing commercially valuable compounds such as citric acid and enzymes, with further potential in pharmaceuticals and biotechnology for protein and secondary metabolite production [14,15,16].

This study aims to explore the potential of cacao mucilage as a growth medium for microbial species, capitalizing on its nutrient-dense composition to support the cultivation of beneficial microorganisms for use in biotechnology and food science.

## 2. Methodology

### 2.1. Experimental Procedure

#### 2.1.1. Obtaining and Characterizing of Cacao Mucilage Powder (CMP)

The cacao mucilage exudate (CME) was obtained from the liquid mucilage of the CCN–51 cacao variety, sourced from local producers in the Santo Domingo de los Tsáchilas province of Ecuador. After 18 h of cacao bean harvesting, the removal of the mucilaginous pulp surrounding the seeds took place. This process involved the enzymatic action of pectin, which led to the liquefaction of the pulp and the subsequent separation of the mucilage from the beans. The drained mucilage then transforms into cacao mucilage exudate (CME) [17]. This step is crucial in cacao processing, as it helps to eliminate the pulp and extract the desired mucilage. The collection process involved using sterile stainless steel containers with a capacity of 10 L, and it was carried out at a temperature of 25 °C. Multiple collections were conducted over up to three days of fermentation, resulting in a total volume of 20 L of liquid mucilage. Once collected, the liquid mucilage was stored at 4 °C until further processing.

To preserve the properties of the mucilage, the CME was subjected to spray drying with a 15% efficiency. The spray-drying process involved a controlled flow rate of 0.1 to 0.5 L per minute. This process was continued until 1.96 kg of powdered mucilage was obtained. The cacao mucilage powder (CMP) obtained from the spray-drying process was stored at 25 °C until it was ready for use [18].

The compositional profile was conducted according to the well-established methods for moisture, protein, fat, fiber, ash, pH, total soluble solids, acidity. Also, phosphorus, potassium, sodium, calcium, and magnesium by in-house ICP/OES method [19,20,21,22,23].

#### 2.1.2. Culture Medium

##### Culture Medium Formulation

The culture medium employed in this study was prepared using commercially available components, and cacao mucilage powder (CMP) obtained as described in Section 2.1.1. Yeast extract (YE), peptone (PEP), and agar–agar (AA) were purchased from Oxoid (Thermo Fisher Scientific, Waltham, MA, USA). The formulation of the culture medium was meticulously established using a mixture design methodology, as elucidated in Section 2.2.1 and outlined comprehensively in Table 1. It is noteworthy that the agar–agar served the specific role of solidifying the medium and was maintained at a constant concentration of 2.5% (*v*/*v*).

To ensure optimal conditions for microbial growth, the pH of each formulation was carefully adjusted to achieve neutral values. The procedure included the initial measurement of pH, in the media suspended in distilled water, with the gradual addition of an acid (HCl) or base (NaOH) while monitoring the pH, until reaching the target pH (neutral). This adjustment was crucial in creating a favorable environment for the microorganisms to thrive and exhibit their growth potential. By standardizing the pH in the different formulations, any variation in microbial growth rates could only be attributed to the specific ingredients and their concentrations, and not to this factor (pH), allowing a full evaluation of the efficacy of the culture medium.

##### Culture Medium Preparation

To create optimal growth environments for the various microbial groups under investigation, specific agar media were meticulously prepared according to the guidelines provided by the manufacturer [24]. The main objective was to tailor the nutrient compositions of the media to support the growth of each target microorganism. For the cultivation of *L. plantarum*, a Man–Rogosa–Sharpe Agar (MRS) was the medium of choice, while Malt Yeast Extract Agar (MYA) was selected to suit the growth of the *S. cerevisiae*. To promote *A. niger* growth, a Sabouraud Dextrose Agar (SDA) was used as a suitable growth medium. All media used were Oxoid brand, and Thermo Fisher Scientific.

The agar medium preparation procedure was methodical and aimed at ensuring the creation of an environment conducive to microbial growth. The process began with the meticulous dissolution of the agar powder in distilled water. Special attention was paid to this step to ensure complete dissolution [25]. The mixture then went through a critical autoclaving phase. This sterilization step was essential to eradicate any possible contaminants, thus safeguarding the purity and integrity of the agar medium [26].

After successful sterilization, the agar medium was carefully poured into sterile Petri dishes. This step required precision to avoid any unintentional contamination that could jeopardize the subsequent microbial culture. Once poured, the medium was allowed to solidify naturally within the Petri dishes. This solidification process was instrumental in creating the proper foundation for microbial growth [27]. The methodology took a comprehensive approach to ensure that the unique nutritional requirements of each microbial group were met through custom agar media. The rigorous preparation process, including dissolution, sterilization, and solidification, was instrumental in establishing a controlled, uncontaminated environment conducive to the successful cultivation of target microorganisms [27].

#### 2.1.3. Microbial Cultivation

##### Preparation of Inoculum

Cell suspensions of three different microbial strains, namely *Saccharomyces cerevisiae*, *Lactiplantibacillus plantarum*, and *Aspergillus niger*, were prepared for enumeration. These strains were chosen based on their relevance to cacao bean fermentation, the focus of the study. The strains were part of the strain collection of the Institute of Food Industry Research (IIIA), where they are preserved and cataloged for use in fermentation studies and other agro-industrial processes. In this regard, pure cultures were obtained through meticulous isolation procedures and maintained under controlled conditions to ensure purity and microbial viability, consistent with standard pre-culture techniques [24]. A small volume of each microorganism was inoculated in Lysogenic Broth (LB medium, Oxoid), which provides the necessary nutrients and conditions for the growth of all microbial groups under study. Once inoculated, each culture is incubated under controlled room temperature conditions (*L. plantarum* 35 °C at 24 h; *S. cerevisiae* 30 °C at 48 h, and *A. niger* 25 °C at 72 h).

For preparation of the cell suspensions, each pure culture was first grown under optimal growth conditions, allowing them to reach a suitable population size. These populations were then diluted to achieve a range of cell concentrations that would be possible to count. The target concentration for each microbial strain was set at 1000 colony-forming units (CFU) per milliliter, a concentration that balances the need for precision against practical feasibility.

To create the necessary dilutions for an accurate count, serial dilution techniques were employed. This involved transferring precise aliquots of the cell suspensions to separate tubes containing sterile diluents, such as distilled water or buffered peptone solution (Merck). Using diluents, the concentration of microbial cells was systematically reduced in a controlled manner, resulting in dilutions of 1:10, 1:100, and 1:1000. These dilutions have been specifically designed to cover a wide range of microbial concentrations, allowing for accurate quantification of microbial load [19]. Serial dilution facilitated accurate counting, ensuring that the counting process was within the range of the counting chamber or slides used for microscopic analysis (avoiding overcrowding of the cells), which could lead to miscounts [25].

##### Inoculation and Incubation

With a sterile pipette, a known volume of 0.1 mL of each dilution was transferred to agar plates containing the previously described media. For yeast and fungi, the inoculum was spread evenly over the surface of the agar plates using a sterile spreader to ensure adequate distribution [24]. For the LAB, the bilayer planting technique was used. In a pipette, a 0.1 mL aliquot of the liquid bacterial culture was gently dispensed onto the surface of the solid agar base layer. The liquid culture was carefully spread to ensure even distribution across a significant area of the agar surface. Subsequently, the liquid culture was allowed to absorb into the agar base layer by leaving the plate undisturbed for a few minutes, facilitating the immobilization of the bacteria within the agar.

A second layer of molten agar, maintained at a temperature of approximately 45–50 °C to avoid thermal damage to the bacteria, was then poured onto the plate, ensuring uniform coverage over the immobilized bacteria [25]. Upon solidification, the resulting plate established a bilayer system where the bacteria were embedded within the solid agar base layer, while the second layer served as an overlay.

To prevent contamination, the Petri dishes were securely sealed with parafilm. Subsequently, the agar plates were placed in an incubator set at the optimal temperature for the growth of each microorganism. *Lactiplantibacillus plantarum* was inoculated on MRS agar and incubated at a temperature range of 30–35 °C for 2–3 days. *S. cerevisiae*, on the other hand, was inoculated on MYA and incubated at a temperature range of 20–25 °C for 2–3 days. For the growth of *Aspergillus niger*, the fungus was inoculated on SDA and incubated at a temperature range of 20–25 °C for a slightly longer duration of 3–7 days. These specific incubation conditions were chosen to ensure optimal growth and development of each microorganism in their respective culture media [25].

### 2.2. Statistical Procedure

#### 2.2.1. Experimental Design

A standard simplex lattice mix design was employed specifically for the selected simplex–lattice (q, m) design. This design involved q components, with q being equal to 3 in this case, allowing for the fitting of a statistical model of order m. The design points encompassed all possible combinations of elements or mixtures, with the proportions represented by xi ranging from 0 to 1 and taking m + 1 values. The specific values of xi were 0, 1/m, 2/m, …, and m/m, corresponding to the proportions in which the components of the mixture participated (x1, x2, …, xq) [27].

The specific design points used were as follows: (x1, x2, x3) = (1, 0, 0); (0, 1, 0); (0, 0, 1); (1/2, 1/2, 0); (1/2, 0, 1/2); and (0, 1/2, 1/2). These design points represented the three pure mixtures and the three binary mixtures, providing a comprehensive range of combinations for the experimental investigation. By utilizing this design, this study aimed to explore the effects of different mixture compositions on the observed outcomes, enabling a thorough analysis of the variables under investigation [28].

The primary objective of implementing this design was to further refine the mathematical models by systematically varying the concentrations of the mixtures to assess the impact of these factors on the growth of microorganisms. This allowed for a more in-depth evaluation of the efficacy of cacao mucilage as a natural culture medium ingredient for microbial development (Table 2).

To increase the size of the experiment, the design was replicated, resulting in a total of 21 mixtures. The formulation consisted of three main ingredients, which together represented sixty percent of the total weight (Table 1): cacao mucilage powder (CMP), yeast extract (YE), and peptone (PEP). The objective was to investigate the effects of these components on the growth rates of the studied microorganisms. The experimental results obtained from the mixture design were adjusted to a quadratic model, represented by the following Equation:

(1) $$E_{\left(y\right)}=\beta_{1}\;*\;x_{1}+\beta_{2}\;*\;x_{2}+\beta_{3}\;*\;x_{3}+\beta_{12}\;*\;x_{1}\;*\;x_{2}+\beta_{13}\;*\;x_{1}\;*\;x_{3}+\beta_{23}\;*\;x_{2}\;*\;x_{3}$$

* Represent multiplication in all equations.

#### 2.2.2. Model Response Variables

The experimental design included the measurement of the recovery rates of three different microbial species: *L. plantarum*, *S. cerevisiae*, and *A. niger.* These recovery rates were selected as the main response variables of interest, reflecting the growth and development of the microorganisms under investigation (Table 3).

To ensure robust and reliable results within the workspace region where maximum microbial growth values are achieved, 21 experimental runs were conducted. This complete experimental setup allowed for thorough exploration and analysis of the variables influencing the recovery rates of the microbial groups. The increased number of experimental runs contributed to enhanced reliability and statistical robustness in the evaluation of microbial growth outcomes.

To enhance the precision and reliability of the experimental results, the allocation of runs in this study was randomized. The values of the blocks, which represented different concentrations of the mixtures used, were determined within the defined study area. This approach, as outlined in Table 2, ensured comprehensive coverage of the experimental space and minimized the potential influence of confounding factors.

#### 2.2.3. Optimization Procedure

##### Numerical and Graphical Optimization

Numerical and graphical optimization was performed with Expert v13 software (Stat–Ease). The objective of the optimization process was to maximize the recovery rates of the three microorganisms by adjusting the proportions of the three ingredients within the specified study range [27,28]. To achieve the maximum objective, the desirability of each response was determined using the following criteria formulas:

(2) $$d_{i}=0,Y_{i}\leq {Low}_{i}$$

(3) $$d_{i}={\left[\frac{Y_{i}-{Low}_{i}}{{High}_{i}-{Low}_{i}}\right]}^{{wt}_{i}},{Low}_{i}<Y_{i}<{High}_{i}$$

(4) $$d_{i}=1,Y_{i}\geq {High}_{i}$$

where d_1_ = 0 represents the minimum desirability, and *d_i_* = 1 represents the maximum desirability.

In the desirability objective function *D*(*x*), each response can be assigned an importance relative to the other responses. The optimization was carried out on the original scale and assigned a maximum importance of five advantages (+++++) to the proposed objective to adjust the desirability function, where importance (ri) varies from the least important (+) value of 1 to the most important (+++++) value of 5 [27]. If varying degrees of importance are assigned to the different responses, the objective function is as follows:

(5) $$D(x)={\left(d_{1}^{r_{1}}\;*\;d_{2}^{r_{2}}\;*\;\ldots \;*\;d_{n}^{r_{n}}\right)}^{\frac{1}{\sum r_{i}}}={\left(\prod_{i=1}^{n}d_{i}^{r_{i}}\right)}^{\frac{1}{\sum r_{i}}}$$

During the graphical optimization of the four responses, the goal was to identify regions where the requirements and critical properties were met simultaneously. This was achieved by overlaying the contours of the critical responses on a contour plot. By doing so, the optimal compromise, or sweet spot, could be selected.

##### Confirmation Analysis

The models obtained from each response were used to provide predictions and interval estimates at the optimization point. During confirmation, the model’s prediction interval was compared to the average of a follow–up sample. If the average of the samples is within the prediction interval, the model is confirmed. Confirmation was made at the most convenient numerical optimization point [28].

To confirm that the model can predict the actual results in the optimal configuration determined from the numerical analysis, seven confirmation runs were performed. The average of those runs is compared to the prediction interval. Smaller intervals indicate good precision in the estimates, and the default value for the prediction interval (L) is 95% = (1 − 0.05) × 100%.

#### 2.2.4. Effect Size Assessment

To evaluate the effectiveness of the formulated mixtures, the effect size was calculated using Cohen’s d (d: 0.2 small; d: 0.5 medium; d: 0.8 large) based on the microbial growth rates [29]. The growth rates achieved in the yeast–extract peptone mucilage (YPM) agar were compared to those obtained in traditional media commonly used for the respective microbial groups.

For *L. plantarum*, the growth rates in the YPM medium were compared to those in Man–Rogosa–Sharpe (MRS) agar. Yeast growth rates in the YPM medium were compared to those in Malt Extract Yeast Extract Glucose Agar (MYA), while the fungal growth rates in the YPM medium were compared to those in Sabouraud Dextrose Agar (SDA). All culture media were from Merck Co. Ltd., Rahway, NJ, USA.

To ensure the reliability and reproducibility of the results, all experiments were conducted in triplicate under identical conditions, using the same concentrations of components and microbial groups. This approach minimized the potential influence of confounding factors and allowed for a robust comparison between the YPM medium and traditional media for each microbial group.

### 2.3. Analytical Determinations

#### 2.3.1. Determination of the Total Microbial Population

To estimate the total number of *L. plantarum* cells in the suspension, a turbidimetric method relying on the McFarland standard was employed. By comparing the turbidity of the bacterial suspension to the turbidity of a well-defined McFarland standard through a standard curve [30], the total number of *L. plantarum* present could be determined. The McFarland density standards were verified using a digital spectrophotometer (Perkin Elmer LAMBDA 1050) with a 1 cm light path at 625 nm. Subsequently, the total number of cells per microliter (Ns) was obtained.

The determination of total yeasts and fungi (spores) was accomplished by direct counting using a Neubauer chamber [31]. The total number of cells per microliter (Ns) of each suspension was calculated from the number of cells counted in the counting area, using the following Equation (6) to convert the count to the number of cells per microliter:

(6) $$Ns=\frac{Ct}{A\;*\;Cd\;*\;Df}$$

where *N* is the number of cells per microliter, *Ct* is the number of cells counted, *A* is the counted area, *Cd* is the chamber depth, and *Df* is the dilution factor.

#### 2.3.2. Determination of Colony-Forming Units (CFU)

Colony-forming units (CFU) were determined using microbial culture techniques in this study. For *S. cerevisiae* and *A. niger*, as well as *L. plantarum*, the serial dilution method was employed [30,31]. Microbial suspensions with known concentrations were cultured using different formulations of the Yeast Extract Peptone Mucilage (YPM) agar, as well as traditional media such as MRS agar, MYA, and SDA.

The microbial cultures were inoculated and incubated according to the procedures outlined in Chapter Inoculation and Incubation. Following incubation, the total cell growth (*N*ν) values were determined using Equation (7). *Nν* represent the number of viable cells. These techniques allowed for the quantification of microbial growth and provided valuable data for comparing the effectiveness of the YPM medium with traditional media in supporting the growth of *L. plantarum*, *S. cerevisiae*, and *A. niger.*

(7) $$Nv=\left(\frac{\frac{1}{n}\sum_{i=1}^{n}{\left(C\right)}_{i}}{V\;*\;p\;*\;d}\right)\;*\;100$$

where *n*: replicas; *i*: the subset of *n*; *C*: the total number of colonies; *V*: Inoculum volume; *p*: number of plates counted; and *d*: minor dilution.

#### 2.3.3. Recovery Rates

The recovery rate (*Rx*) for each microbial group was determined by evaluating the *Nv* (number of viable cells) within each group relative to the total number of microorganisms in the corresponding microbial suspension, indicated as *Ns*. To calculate recovery rates for *L. plantarum, S. cerevisiae*, and *A. niger*, Equation (8) was used, which provides specific formulas for each microbial group. The total recovery rate (RR) was calculated as the average of the recovery rates for each microbial group, as expressed in Equation (9).

In the context of the mixture design, the recovery rates obtained for each microbial group (*Rx*) and the total recovery rate (*RR*) were considered response variables. These response variables served as crucial indicators to assess the efficacy of the formulated medium. Additionally, parallel evaluations were performed using traditional media to compare the recovery rates obtained on the optimized media. This analysis aimed to determine the size of the effect, thus providing information on optimized media for promoting microbial growth and recovery.

(8) $$R(x)=\left(\frac{\frac{1}{n}\sum_{i=1}^{n}{\left(Nv\right)}_{i}}{\frac{1}{n}\sum_{i=1}^{n}{\left(Ns\right)}_{i}}\right)\;*\;100$$

where *x* is the microbial group, *i*: replicas, *n*: a total of replicas, *Nv*: the number of viable cells per milliliter, and *Ns*: the total number of cells per milliliter in suspension.

(9) $$RR=\frac{1}{3}\sum R(x)$$

where *R* is the recovery rate, and *x* is the microbial group.

## 3. Results

### 3.1. Compositional Profile of Cacao Mucilage

The raw cacao mucilage used in this study exhibited a high moisture content of 85.83 ± 0.14%, a pH of 3.41 ± 0.03, total soluble solids of 14.65 ± 0.10 °Brix, and an acidity of 0.72 ± 0.03% (citric acid). The content of protein was 3.75 ± 0.12%, lipids of 3.21 ± 0.10%, fiber of 9.51 ± 0.3% and ash of 3.11 ± 0.90%. On the other hand, the content of phosphorus, potassium, sodium, calcium, and manganese were 89.10 ± 4.3, 89.10 ± 4.3, 33.47 ± 4.40, 205.05 ± 44.00, and 79.81 ± 0.70 mg/kg, respectively.

### 3.2. Analysis of the Obtained Models

Table 3 presents the recovery rates obtained for each microorganism through different mixtures. Among these mixtures, formulations 8, 12, 14, 15, 17, 18, and 20 demonstrated the highest recovery rate of *S. cerevisiae*, reaching 96.65%. Similarly, formulations 8, 14, 15, 17, and 20 exhibited the highest recovery rate of *L. plantarum*, with a value of 97.44%. In addition, it was found that the recovery rate of *A. niger* was higher in formulations 6, 9, and 19. However, this microorganism exhibited the lowest recovery rates. These findings indicate cacao mucilage’s effectiveness in promoting the growth and recovery of the study microorganisms.

The mixture design analysis yielded four significant models (Table 4) that effectively describe the relationship between recovery rates of different microorganisms (R1: *S*. *cerevisiae*, R2: *L*. *plantarum*, R3: *A*. *niger*, and R4: total) and the proportions of various ingredients (A: mucilage, B: yeast extract, C: peptone). These models exhibited a statistically significant influence (*p* < 0.05) on the studied variables, with a confidence level of 95%. Specifically, the mixtures of mucilage/yeast extract (AB) and mucilage/peptone (AC) were consistently identified as significant terms (*p* < 0.05) in all models.

Furthermore, in the R2 model, the yeast extract/peptone (BC) mixture also demonstrated significance (*p* < 0.05). Moreover, for the recovery rates of R3 and R4, the combination of all three components (ABC) in the mixture was found to be significant (*p* < 0.05) (Table 4). These results emphasize the importance of the specific ingredient combinations that influence the recovery rates of different microorganisms, highlighting the significance of optimizing the proportions of mucilage, yeast extract, and peptone in promoting microbial recovery.

The lack of fit tests conducted on the four models revealed non-significant results (*p* > 0.05), indicating that these models adequately capture the variation observed between replicates without disregarding data due to random error (Table 4). The predicted and fitted coefficients of determination (R^2^) for each model exceeded 0.5, and the predicted R^2^; values closely aligned with the fitted R^2^ values, differing by less than 0.2 [27,28]. Hence, these models provide a reliable framework for exploring the design space (Table 4). Each mathematical model effectively represents the expected change in the response variable per unit change in the corresponding variable, while maintaining the other variables constant [27].

### 3.3. Microbial Species’ Recovery Rate

**Figure 1:**
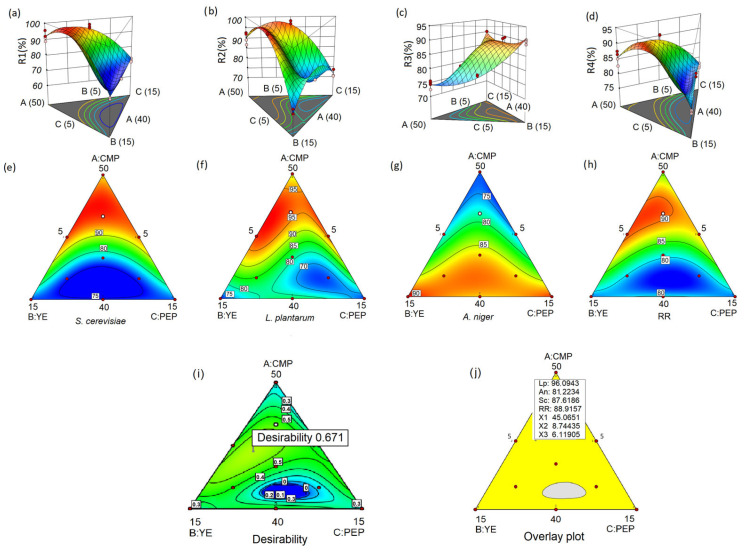
Response surface and contour of recovery rate. R1: *S. cerevisiae* (**a**,**e**); R2: *L plantarum* (**b**,**f**); R3: *A. niger* (**c**,**g**); and R4: RR (**d**,**h**). The contour plot of maximum desirability (**i**) and overlaid graph represented by different regions indicating maximum (yellow areas) and minimum (gray areas) (**j**). The optimum values of the ingredients and the four recovery rates are shown as a flag planted. The gradient colors (red-green-blue) indicate different levels of responses: red indicates areas with the highest values, green represents intermediate values, and blue indicates areas with the lowest values. Contour lines delimit areas with the same response value, with each line corresponding to a constant value, allowing to visualize how responses vary within the experimental space.

Figure 1a,e display the recovery rate of *S*. *cerevisiae*. The contour plots and response surface generated from the models reveal a clear pattern, indicating that an increase in mucilage concentration corresponds to a higher recovery rate of *S*. *cerevisiae*, as evidenced by the prominent red areas in the plots. The observed positive relationship between mucilage concentration and the yeast recovery rate can primarily be attributed to the significant mixtures of mucilage/peptone and mucilage/yeast extract, as indicated by the statistical analysis (Table 4). Conversely, when the concentration of mucilage in the mixtures decreases, there is a marked decrease in the recovery rate of *S*. *cerevisiae*.

The contour plot and response surface analysis (Figure 1b,f) reveal a similar pattern in the recovery rate of *L*. *plantarum* compared to *S*. *cerevisiae*. Increasing the concentration of cacao mucilage in the mixture leads to a noticeable improvement in the recovery rate of *L*. *plantarum*. Furthermore, evidence suggests that decreasing the proportion of yeast extract while simultaneously increasing the mucilage concentration significantly enhances the recovery rate of *L*. *plantarum*.

However, a distinct crest is observed in the response surface plot for *L*. *plantarum*, indicating a potential limit to the beneficial effects of mucilage concentration on its recovery rate. This suggests that there may be an optimal range of mucilage concentration that yields the desired effect for *L*. *plantarum* (Figure 1b,f).

In contrast to *S*. *cerevisiae* and *L. plantarum*, the recovery rate of *A*. *niger* exhibited a distinct response to variations in mucilage, yeast extract, and peptone concentrations in the mixtures (Figure 1c,g). Specifically, as the mucilage concentration increases, the recovery rate of *A*. *niger* decreases. Conversely, an increase in the proportions of yeast extract and peptone in the mixtures resulted in an improvement in the recovery rate of *A*. *niger*.

The graphical representation suggests that optimizing the recovery rate of *A*. *niger* requires targeting specific values beyond designated limits, where maximum recovery rates can be achieved. To maximize the recovery rate of *A*. *niger*, adjustments to the proportions of the design can be made.

The study findings underline the importance of strategic manipulation of formulation components to optimize the recovery rate of *L*. *plantarum*, *S*. *cerevisiae*, and *A*. *niger*. Increasing the mucilage concentration while reducing the proportions of yeast extract and peptone is predicted to have a positive impact on the growth and recovery of bacteria and yeast in the given context. Furthermore, the results emphasize the importance of lower concentrations of mucilage and high concentrations of yeast extract and peptone to achieve optimal recovery of the fungus.

These findings provide valuable information to optimize the recovery process of these microbial species and underscore the crucial role of cacao mucilage in recovery and growth promotion.

The observations presented in Figure 1d,h provide valuable insights into the behavior of the total recovery rate and its relation to the mucilage concentration. It becomes apparent that as the mucilage concentration increases significantly, there is a noticeable upward trend in the total recovery rate. This finding strongly suggests that higher concentrations of mucilage have the potential to contribute to improved recovery rates, which in turn opens the possibility of reducing the proportions of yeast extract and peptone in the formulation while still achieving desirable recovery results.

The implications of these findings are significant. By carefully manipulating the mucilage concentration, it becomes feasible to optimize the full recovery rates of the microbial groups under investigation. This observation serves as compelling evidence that the inclusion of mucilage in the culture medium plays a crucial role in enhancing the recovery rates of these microorganisms.

### 3.4. Optimization Results

The visualization of the superimposed graph in Figure 1i reveals the optimization process of the four response variables. Within this context, an area with an optimal formulation characterized by specific coordinates where the maximum recovery rates of all microbial groups are obtained is identified.

Analysis of the specific coordinates of the optimal formulation indicated as X1: A, X2: B, and X3:C reveals that the optimal formulation consists of 44.53% mucilage (A), 9.05% yeast extract (B), and 6.40% peptone (C). It is noteworthy that within these coordinates, the four response variables that represent recovery rates (R1, R2, R3, and R4) reach their highest values (Figure 1e–h).

The recovery rates achieved by the optimized formulation are remarkable for *S. cerevisiae*, with a recovery rate of 87.60%, while *L. plantarum* demonstrates a recovery rate of 96.09%, and *A. niger* presents a recovery rate of 82.70%. Taking all microbial groups into account, the overall recovery rate reaches 87.40% (Figure 1i).

These compelling results provide strong evidence for the efficacy of the optimized formulation. The achievement of maximum recovery rates for all microbial groups within the identified coordinates emphasizes the success of the optimization process and its possible practical applications. These findings underscore the crucial role that mucilage, yeast extract, and peptone play in promoting the growth and recovery of *L. plantarum*, *S. cerevisiae*, and *A. niger*, as well as the efficiency of the overall process.

The evaluation of the experimental formulation at the point of optimization, as presented in Table 5, highlights the robustness of the model’s predictions. With a confidence level of 95%, the average observations of each confirmation experiment align closely with the prediction interval of the confirmation node. This alignment demonstrates the accuracy and reliability of the model’s predictions at the specified coordinates, as depicted in Figure 1.

The desirability value indicated by a flag (0.671) in Figure 1i is also the overlap point in Figure 1j where the recovery rates for the three microorganisms (*L. plantarum*: 96.09, *A. niger*: 81.22, and *S. cerevisiae*: 87.61) are shown. The presence of large green zones in the desirability analysis (Figure 1i) to maximize the recovery of the three microorganisms indicates that there is a wide range of combinations of mucilage, peptone, and yeast extract that allows obtaining high recovery percentages (Figure 1j). This suggests that the system is robust and flexible, since small variations in the proportions of the components do not significantly affect the results. This feature highlights the effectiveness of cacao mucilage as a base of the culture medium, providing favorable conditions for the growth and recovery of the microorganisms of study in a wide range of formulations.

The successful validation of the model’s predictions through confirmatory experiments reinforces the practicality and effectiveness of the optimized formulation. The confirmed predictions support the suitability and reproducibility of the identified formulation coordinates, which involve specific ratios of mucilage, yeast extract, and peptone. These findings assure that the optimized formulation can consistently yield the desired outcomes and provide a reliable foundation for further experimentation and practical applications.

Effect size analysis, as presented in Table 6, sheds light on the impact of optimized YPM medium compared to traditional culture media on microbial growth. When examining the growth values of *S. cerevisiae* and *L. plantarum* in the traditional media (MRS, MYA, and SDA) versus the YPM medium, it should be noted that the traditional media generally exhibited higher growth values. However, these growth differences were found to not be statistically significant (*p* > 0.05) for both *S. cerevisiae* and *L. plantarum*. The analysis of the size of the effect, expressed through the d–Cohen values, also indicates that the magnitude of these differences is relatively low (0.2) for both microbial groups. Dispersion of data for the YMP medium suggests a broader range of underlying factors or influences that contribute to variation in microbial growth.

On the contrary, a significant difference (*p* < 0.05) was observed in the fungal growth values between the YPM medium and the SDA medium. The effect size analysis, represented by a Cohen d value greater than 0.8, underscores a substantial effect size. This means that the disparities in fungal growth between these two media are considerable, with the YPM medium showing a clear disadvantage.

Although traditional media may exhibit slightly higher growth values for *S. cerevisiae* and *L. plantarum*, the lack of statistical significance and relatively low effect sizes suggest that the optimized YPM medium remains a viable alternative. Furthermore, the significant disparity in fungal growth between YPM medium and SDA medium underscores the importance of optimizing the formulation to promote optimal fungal growth. These findings contribute to a deeper understanding of the differential effects of the YPM medium on various microbial groups and emphasize the need for further research and refinement to improve its overall performance.

## 4. Discussion

A mixed design was employed to assess the collective behavior of various factors impacting microbial recovery rates, shedding light on the individual contributions of each factor. Notably, an increase in mucilage concentration exhibited a profound influence on the recovery rates of *S. cerevisiae*, *L. plantarum*, and *A. niger*, resulting in substantial improvements. This scientific discussion explores the underlying mechanisms driving these observations and their broader implications.

### 4.1. Cacao Mucilage Acts as a Catalyst for Microbial Growth

Microbial behavior within complex mixtures, such as cocoa mucilage, is shaped by their nutritional requirements and the specific composition of the substrate. Cacao mucilage provides an optimal medium due to its high concentrations of glucose, fructose, essential vitamins, nitrogen, and trace elements, all of which meet the metabolic needs of microorganisms like Saccharomyces *cerevisiae*, *Lactiplantibacillus plantarum*, and *Aspergillus niger* [9,10,11,12,13]. Research has consistently demonstrated a positive correlation between elevated sugar levels, particularly glucose and fructose, and enhanced microbial growth in cacao mucilage, as these sugars serve as primary carbon sources essential for microbial proliferation [32,33,34,35,36].

Microorganisms possess specific metabolic capacities to utilize the nutritional components of cacao mucilage, with consistent manipulation of mucilage concentrations yielding similar effects on microbial growth, underscoring the importance of sugars as a key nutrient source. Among these microbes, *S. cerevisiae*, a prominent cacao yeast, efficiently converts glucose and fructose into alcohol and flavor compounds, while lactic acid bacteria like *L. plantarum* metabolize these sugars to produce lactic acid, contributing to the fermentation’s acidity [33,34,35]. Additionally, for fungi such as *A. niger*, glucose and fructose are essential for growth and enzymatic activity, including the induction of conidial germination, which plays a vital role in the fermentation process [37].

### 4.2. S. cerevisiae and L. plantarum Rely on Higher Concentrations of Pure Mucilage for Optimal Growth

The study’s findings highlight the significance of specific nutrient concentrations in supporting the growth of yeasts in mucilage. Particularly, higher concentrations of pure, unmixed mucilage are shown to nurture yeast growth effectively. This parallels the reliance of both yeasts and LAB on increased sugar concentrations, particularly glucose and fructose, for their growth and metabolic activities. *S. cerevisiae*, renowned for its adeptness in fermenting sugars, exemplifies this, utilizing glycolysis to break down glucose and fructose, generating energy in the form of ATP and precursor molecules crucial for organic compound synthesis [35,36,37,38].

The importance of glucose and fructose to *S. cerevisiae* and *L. plantarum* during cacao fermentation is consistently underscored, as these sugars are pivotal for their growth and metabolism. Intriguingly, the utilization of glucose and fructose from mucilage unfolds dynamically [35]. Glucose is swiftly consumed within the initial 48 to 72 h of fermentation, followed by the prominence of fructose utilization after approximately 120 h [35,36,37]. This preferential glucose uptake by yeasts can convert sucrose to shape the microbial process and life cycle dynamics over time. These findings emphasize the positive impact of mucilage on these microorganisms and their functions, unveiling the intricate interplay of nutrients in the cacao fermentation process.

### 4.3. High Levels of PEP and YE Are Essential for Optimal Growth of A. niger

The results indicate that by increasing the mucilage concentration, YE and PEP can potentially be reduced without compromising the RR. However, the recovery of *A. niger* depends more on high concentrations of PEP and YE for optimal growth. The study’s findings suggest that an increase in cacao mucilage concentration could lead to a reduction in the requirements for yeast extract (YE) and peptone (PEP) without compromising microbial recovery. However, optimal growth of *A. niger* appears to depend more on higher concentrations of PEP and YE.

Traditionally, yeast extract and peptone have been used to meet the nutritional needs of microorganisms [39]. In the designed formulation, it compensates for the low nitrogen content of the mucilage, making it a suitable culture medium for *S. cerevisiae*, *L. plantarum*, and *A. niger*.

Yeast extract and peptone, derived from yeast cells and animal tissues, respectively, effectively assimilate nitrogenous compounds, amino acids, and peptides, fulfilling the microorganism’s nitrogen requirements [39,40]. This strategy enhances nitrogen source utilization, stimulating the multiplication of yeasts, LAB, and fungi essential for enzyme, coenzyme, nucleic acid, vitamin, and cellular component synthesis [41]. The greater dependence of *A. niger* on nitrogen sources for growth compared to *S. cerevisiae* and *L. plantarum* is attributed to factors such as its expansive filamentous structures, enabling efficient nutrient uptake, metabolic versatility in utilizing diverse nitrogen sources, and nitrogen’s critical role in fungal growth processes [42]. In contrast, unicellular yeasts and bacteria depend on mechanisms like diffusion or active transport for nutrient acquisition, which might limit their nitrogen uptake efficiency compared to fungi [43].

### 4.4. A. niger Shows Limited Growth at High Concentrations of Mucilage

Fungi’s susceptibility to osmotic stress significantly impacts their growth inhibition in concentrated sugar solutions, setting them apart from yeasts and lactic acid bacteria (LABs) [44,45,46]. This distinction arises from multiple factors, encompassing the distinctive structural and physiological attributes of fungal membranes, the repercussions of osmotic imbalances on cellular water equilibrium, and the differential adaptations of yeasts and LABs to osmotic stress [47,48,49,50,51].

Primarily, fungal membranes, enriched with ergosterol, exhibit higher vulnerability to osmotic stress compared to yeasts and LABs [48]. The composition and structure of ergosterol-rich membranes confer increased fluidity and permeability, rendering them more susceptible to damage under osmotic stress [49]. In contrast, yeast and LAB membranes’ composition imparts greater stability and resistance against osmotic imbalances. Furthermore, the presence of concentrated sugar solutions, like those in mucilage, can trigger osmotic imbalance affecting cellular water content. Fungal cells, facing such solutions, encounter a net water loss due to higher osmotic pressure externally [49,50]. This loss leads to cell dehydration, significantly impairing fungal growth and viability, and disrupting water uptake and retention.

In contrast, yeasts and LABs have evolved robust osmoregulation mechanisms to endure osmotic stress and flourish in high-sugar environments [51,52]. Yeasts utilize high–affinity sugar transporters and osmotic stress-responsive signaling pathways, adapting to high-sugar conditions [53,54]. LABs showcase resilient membrane integrity and defensive mechanisms safeguarding against osmotic stress-induced harm [53,54,55,56,57].

### 4.5. The Effect Size Indicates That the YPM Medium Is Comparable to Its Traditional Counterparts

The YPM medium demonstrates comparable growth potential to traditional media for *S. cerevisiae* and *L. plantarum*, as indicated by non-significant differences in growth values. The effect size analysis further supports this, suggesting minimal practical differences. The optimized composition of YPM, including nutrient composition and growth-promoting factors, makes it a viable alternative for yeast and LAB cultures.

However, contrasting results were observed in fungal growth between YPM and SDA media. A significant difference in fungal growth values was noted, indicating a substantial impact of the medium type on fungal growth. This was reinforced by a large effect size (Cohen’s d > 0.8), underlining the significant disparities in fungal growth between the two media.

These disparities emphasize the necessity for ongoing optimization of YPM media, especially to better accommodate the requirements of fungi. While the YPM medium holds promise for yeast and LAB cultures, further adjustments are needed, potentially involving ingredient modifications, pH adjustments, and tailored growth factors to better suit the specific needs of fungal growth.

### 4.6. Future Contributions of the YPM Medium and Mucilage

The YPM medium has demonstrated comparable growth efficacy to conventional media for *Saccharomyces cerevisiae* and *Lactiplantibacillus plantarum*, indicating its potential as a viable culture medium. Further refinement of YPM composition, such as optimizing ingredient ratios, pH, and adding specific growth factors, could expand its use across a broader microbial spectrum, particularly for fungal species. Continued research is essential to adapt YPM to broader applications, enhancing its versatility in microbial biotechnology.

Studies highlight that microorganisms associated with cacao mucilage can produce bioactive compounds with antimicrobial and enzymatic properties, positioning cacao mucilage as a promising substrate for biotechnological applications [4,5,6]. The potential of cacao mucilage-based media to facilitate the production of secondary metabolites, including antimicrobials, antioxidants, enzymes, and polyphenols, offers valuable applications in the pharmaceutical, nutraceutical, and cosmeceutical sectors.

In addition to bioactive production, cacao mucilage also supports microbial biodegradation, utilizing sugars, organic acids, and phenolic compounds to enhance the breakdown of complex contaminants such as hydrocarbons and pesticides. These properties can promote sustainable bioprocessing to produce biopolymers, packaging, and biomedical materials and further offer potential environmental benefits through remediation. By fostering beneficial microbial interactions, improving soil structure, and increasing biomass, cacao mucilage underlines its versatile applications in the pharmaceutical, environmental, industrial, and agricultural sectors.

## 5. Conclusions

This study provides a strong foundation for optimizing the YPM microbial culture medium, demonstrating the key influence of mucilage concentration in improving microbial recovery rates. The mucilage’s protective, adhesive, and nutritive qualities play a significant role in boosting recovery efficiency. These results pave the way for new biotechnological applications, showcasing cacao mucilage as a flexible and sustainable medium for future research and industrial advancements.

This study focused on the design of the experimental space and specific microbial cultures. Future research should consider exploring other regions beyond the experimental design space, evaluating other microbial cultures from various sources, and identifying key growth factors, to refine culture media formulations. Furthermore, with the identification of potential growth strategies based on specific nutritional needs, even higher recovery rates could be achieved in different microbial species.

## Figures and Tables

**Table 1:** Mix design components of the culture medium.

Labels						Central Tendency Measures	
Factors	Variables	Minimum	Maximum	Minor Code	High Code	Mean	Sd
A	CMP	40	50	+0 ↔ 40	+0.5 ↔ 50	43.63	3.83
B	YE	5	15	+0 ↔ 5	+0.5 ↔ 15	8.02	3.67
C	PEP	5	15	+0 ↔ 5	+0.5 ↔ 15	8.25	3.63

CMP: Cacao mucilage powder; YE: yeast extract; PEP: peptone; Sd: standard deviation.

**Table 2:** Experimental runs and mixture design results.

Random Runs	Blocks	Runs	CMP	YE	PEP
3	Block 1	1	50	5	5
21	Block 1	2	50	5	5
11	Block 1	3	43.33	8.33	8.33
19	Block 1	4	40	5	15
2	Block 1	5	40	5	15
12	Block 1	6	40	15	5
7	Block 1	7	41.66	6.66	11.66
10	Block 1	8	46.66	6.66	6.66
9	Block 1	9	40	5	15
18	Block 1	10	40	15	5
13	Block 1	11	40	15	5
8	Block 2	12	50	5	5
17	Block 2	13	40	10	10
15	Block 2	14	45	5	10
1	Block 2	15	45	10	5
4	Block 2	16	40	10	10
16	Block 2	17	45	5	10
5	Block 2	18	45	5	10
20	Block 2	19	41.66	11.66	6.66
14	Block 2	20	45	10	5
6	Block 2	21	50	5	5

CMP: cacao mucilage powder; YE: yeast extract; PEP: peptone.

**Table 3:** Microbial recovery rates in the formulations of culture mediums.

Runs	CEP	YE	PEP	R1(%)	R2(%)	R3(%)	R4(%)
1	50	5	5	88.74	86.72	72.42	82.627
2	50	5	5	91.91	91.9	75.53	86.447
3	43.33	8.33	8.33	73.35	83.78	84.64	80.590
4	40	5	15	71.03	70.55	87.77	76.450
5	40	5	15	72.52	70.31	88.58	77.137
6	40	15	5	71.25	70.61	90.6	77.487
7	41.66	6.66	11.66	70.9	72.71	86.43	76.680
8	46.66	6.66	6.66	96.22	94.77	77	89.330
9	40	5	15	70.16	73.73	90.15	78.013
10	40	15	5	70.28	70.96	88.95	76.730
11	40	15	5	70.25	73.27	89.99	77.837
12	50	5	5	95.56	92.55	74.33	87.480
13	40	10	10	70.37	89.56	85.56	81.830
14	45	5	10	97.07	98.06	76.03	90.387
15	45	10	5	98.41	98.7	78.13	91.747
16	40	10	10	74.93	77.44	89.67	80.680
17	45	5	10	95.52	96.68	78.53	90.243
18	45	5	10	94.81	93.95	75.28	88.013
19	41.66	11.66	6.66	72.87	77.56	90.36	80.263
20	45	10	5	97.12	99.02	77.68	91.273
21	50	5	5	90.89	89.09	74.98	84.987

CMP: cacao mucilage powder; YE: yeast extract; PEP: peptone; R1: *S. cerevisiae* rate; R2: *L. plantarum* rate; R3: *A. niger* rate; R4: total rate.

**Table 4:** ANOVA and fit statistics results of mixture design.

Models	Sig. Term *p* < 0.05	Lack of Fit (*p*)	F-Value	*p* Value	R^2^ Adjusted	R^2^ Predicted	CV (%)	Adeq Precision
Model R1	AB, AC	0.56	84.43	<0.00	0.97	0.93	2.14	22.48
Model R2	AB, AC, BC	0.19	15.57	<0.00	0.82	0.65	4.32	11.59
Model R3	AB, AC ABC	0.98	52.14	<0.00	0.94	0.88	1.86	17.59
Model R4	AB, AC ABC	0.39	57.39	<0.00	0.92	0.81	1.51	16.68

Model terms with *p*-values below 0.05 are considered statistically significant, indicating their importance in the model. Non-significant terms are removed, resulting in reduced models. The equations of these reduced models are expressed as functions of the coded terms. The lack of fit of the models is non-significant when compared to the pure error, suggesting that the models adequately fit the data. The Predicted R^2^ is in reasonable agreement with the Adjusted R^2^, i.e., the difference is less than 0.2. CV: variation coefficient.

**Table 5:** Predicted results.

Responses	Predicted Mean	Standard Deviation	Standard Error	Number of Runs	Lower Limit LI	Observed Mean	Upper Limit. LS
R1	87.607	1.771	1.680	7	83.920	88.160	91.316
R2	94.280	3.700	2.357	7	89.187	91.191	99.372
R3	82.705	1.537	1.111	7	80.303	81.742	85.103
R4	87.399	1.252	1.187	7	84.788	87.897	90.018

**Table 6:** Effect size: Cohen’s t and d test.

M.O	R (%)	YPM (UFC/g)	MRS (UFC/g)	MYA (UFC/g)	SDA (UFC/g)	d–Cohen	*p*
*S. cerevisiae*	88.160	7.68 ± 5.95 ^a^	–	8.73 ± 0.62 ^a^	–	0.2	0.649
*L plantarum*	91.191	7.74 ± 6.08 ^a^	8.84 ± 0.62 ^a^	–	–	0.2	0.642
*A. niger*	81.742	7.31 ± 1.46 ^a^	–	–	9.16 ± 0.68 ^b^	1.6	0.001

MRS: Man–Rogosa–Sharpe Agar; MYA: Malt Yeast Extract Agar; SDA: Sabouraud Dextrose Agar; YPM: yeast extract peptone mucilage. Statistical differences expressed in superscript letters are determined in rows.

## Data Availability

The original contributions presented in this study are included in the article. Further inquiries can be directed to the corresponding author.

## References

[1] Bastidas J.V., Badillo Melo W.A., Briones-Bitar J. (2022). Sustainability of the Cocoa Industry: Cocoa Waste Mucilage Use to Produce Fermented Beverages. Case Study in Los Ríos Province. Int. J. Sustain. Dev. Plan..

[2] Balladares C., Garca J., ChezGuaranda I., Prez S., Gonzlez J., Sosa D., Viteri R., Barragn A., QuijanoAviles M., Manzano P. (2016). Physicochemical Characterization of Theobroma Cacao L. Mucilage, in Ecuadorian Coast. Emir. J. Food Agric..

[3] Chicaiza Intriago J.G., Zambrano Briones G.E., Delgado Villafuerte C.R., Ávila Martínez M.F., Pincay Cantos M.F. (2024). Linear Correlation Analysis of Production Parameters of Biofuel from Cacao (*Theobroma cacao* L.) Mucilage. J. Ecol. Eng..

[4] Llerena W., Samaniego I., Vallejo C., Arreaga A., Zhunio B., Coronel Z., Quiroz J., Angós I., Carrillo W. (2023). Profile of Bioactive Components of Cocoa (*Theobroma cacao* L.) By-Products from Ecuador and Evaluation of Their Antioxidant Activity. Foods.

[5] Martínez R., Torres P., Meneses M.A., Figueroa J.G., Pérez-Álvarez J.A., Viuda-Martos M. (2012). Chemical, Technological and in Vitro Antioxidant Properties of Cocoa (*Theobroma cacao* L.) Co-Products. Food Res. Int..

[6] Vargas-Arana G., Merino-Zegarra C., Tang M., Pertino M.W., Simirgiotis M.J. (2022). UHPLC–MS Characterization, and Antioxidant and Nutritional Analysis of Cocoa Waste Flours from the Peruvian Amazon. Antioxidants.

[7] Morante-Carriel L., Abasolo F., Bastidas-Caldes C., Paz E.A., Huaquipán R., Díaz R., Valdes M., Cancino D., Sepúlveda N., Quiñones J. (2023). Isolation and Characterization of Lactic Acid Bacteria from Cocoa Mucilage and Meat: Exploring Their Potential as Biopreservatives for Beef. Microbiol. Res..

[8] Ghisolfi R., Bandini F., Vaccari F., Bellotti G., Bortolini C., Patrone V., Puglisi E., Morelli L. (2023). Bacterial and Fungal Communities Are Specifically Modulated by the Cocoa Bean Fermentation Method. Foods.

[9] Parapouli M., Vasileiadi A., Afendra A.-S., Hatziloukas E. (2020). *Saccharomyces cerevisiae* and Its Industrial Applications. AIMS Microbiol..

[10] Narendra K.S. (2023). Lactobacillus Plantarum: A Potential Health Booster—A Comprehensive Review. Int. J. Sci. Res. Eng. Manag..

[11] Lee Y., Jaikwang N., Kim S.K., Jeong J., Sukhoom A., Kim J.-H., Kim W. (2023). Characterization of a Potential Probiotic *Lactiplantibacillus plantarum* LRCC5310 by Comparative Genomic Analysis and Its Vitamin B_6_ Production Ability. J. Microbiol. Biotechnol..

[12] Chen Q., Liu B., Liu G., Shi H., Wang J. (2023). Effect of *Bacillus subtilis* and *Lactobacillus plantarum* on Solid-state Fermentation of Soybean Meal. J. Sci. Food Agric..

[13] Behera B.C. (2020). Citric Acid from *Aspergillus niger*: A Comprehensive Overview. Crit. Rev. Microbiol..

[14] Lin W., Xu X., Lv R., Huang W., ul Haq H., Gao Y., Ren H., Lan C., Tian B. (2021). Differential Proteomics Reveals Main Determinants for the Improved Pectinase Activity in UV-Mutagenized *Aspergillus niger* Strain. Biotechnol. Lett..

[15] Conesa A., van den Hondel C.A., Punt P.J. (2000). Studies on the Production of Fungal Peroxidases in *Aspergillus niger*. Appl. Environ. Microbiol..

[16] Vergara-Mendoza M., Martínez G.R., Blanco-Tirado C., Combariza M.Y. (2022). Mass Balance and Compositional Analysis of Biomass Outputs from Cacao Fruits. Molecules.

[17] Cervantes-Martínez C.V., Medina-Torres L., González-Laredo R.F., Calderas F., Sánchez-Olivares G., Herrera-Valencia E.E., Gallegos Infante J.A., Rocha-Guzman N.E., Rodríguez-Ramírez J. (2014). Study of Spray Drying of the Aloe Vera Mucilage (*Aloe vera barbadensis* Miller) as a Function of Its Rheological Properties. LWT-Food Sci. Technol..

[18] Weenk G.H. (1992). Microbiological Assessment of Culture Media: Comparison and Statistical Evaluation of Methods. Int. J. Food Microbiol..

[19] AOAC (2000). Protein (Crude) in Animal Feed, Forage (Plant Tissue), Grain, and Oil Seeds. Block Digestion Method Using Copper Catalyst and Steam Distillation into Boric Acid.

[20] AOAC (2000). Fat (Crude) or Ether Extract in Animal Feed.

[21] AOAC (2000). Crude Fiber in Cacao Products.

[22] AOAC (2000). Ash (Acid-Insoluble) of Cacao Products.

[23] AOAC (2000). Loss on Drying (Moisture) in Plants.

[24] (2017). Microbiology of the Food Chain—Preparation of Test Samples, Initial Suspension and Decimal Dilutions for Microbiological Examination.

[25] Bonnet M., Lagier J.C., Raoult D., Khelaifia S. (2020). Bacterial Culture through Selective and Non-Selective Conditions: The Evolution of Culture Media in Clinical Microbiology. New Microbes New Infect..

[26] Abdel Massih M., Planchon V., Pitchugina E., Mahillon J. (2019). Enumeration of Lactic Acid Bacteria: Lacuna and Improvement Areas Highlighted by Proficiency Testing. Accredit. Qual. Assur..

[27] Galvan D., Effting L., Cremasco H., Conte-Junior C.A. (2021). Recent Applications of Mixture Designs in Beverages, Foods, and Pharmaceutical Health: A Systematic Review and Meta-Analysis. Foods.

[28] Gunst R.F. (1996). Response Surface Methodology: Process and Product Optimization Using Designed Experiments. Technometrics.

[29] Maher J.M., Markey J.C., Ebert-May D. (2013). The Other Half of the Story: Effect Size Analysis in Quantitative Research. CBE—Life Sci. Educ..

[30] Wang R., Lorantfy B., Fusco S., Olsson L., Franzén C.J. (2021). Analysis of Methods for Quantifying Yeast Cell Concentration in Complex Lignocellulosic Fermentation Processes. Sci. Rep..

[31] Downey A.S., Da Silva S.M., Olson N.D., Filliben J.J., Morrow J.B. (2012). Impact of Processing Method on Recovery of Bacteria from Wipes Used in Biological Surface Sampling. Appl. Environ. Microbiol..

[32] Vizcaino-Almeida C.R., Guajardo-Flores D., Caroca-Cáceres R., Serna-Saldívar S.O., Briones-García M., Lazo-Vélez M.A. (2022). Non-conventional Fermentation at Laboratory Scale of Cocoa Beans: Using Probiotic Microorganisms and Substitution of Mucilage by Fruit Pulps. Int. J. Food Sci. Technol..

[33] Viesser J.A., de Melo Pereira G.V., de Carvalho Neto D.P., Rogez H., Góes-Neto A., Azevedo V., Brenig B., Aburjaile F., Soccol C.R. (2021). Co-Culturing Fructophilic Lactic Acid Bacteria and Yeast Enhanced Sugar Metabolism and Aroma Formation during Cocoa Beans Fermentation. Int. J. Food Microbiol..

[34] Da Veiga Moreira I.M., Miguel M.G.d.C.P., Duarte W.F., Dias D.R., Schwan R.F. (2013). Microbial Succession and the Dynamics of Metabolites and Sugars during the Fermentation of Three Different Cocoa (*Theobroma cacao* L.) Hybrids. Food Res. Int..

[35] Garcia Gonzalez E., Ochoa Muñoz A.F., Montalvo Rodríguez C., Ordoñez Narvaéz G.A., Londoño Hernández L. (2021). Sucesión Microbiana Durante La Fermentación Espontánea de Cacao En Unidades Productivas. Cienc. Desarro..

[36] Lima C.O.D.C., Vaz A.B., De Castro G.M., Lobo F., Solar R., Rodrigues C., Martins Pinto L.R., Vandenberghe L., Pereira G., Miúra da Costa A. (2021). Integrating Microbial Metagenomics and Physicochemical Parameters and a New Perspective on Starter Culture for Fine Cocoa Fermentation. Food Microbiol..

[37] Ijadpanahsaravi M., Punt M., Wösten H.A.B., Teertstra W.R. (2021). Minimal Nutrient Requirements for Induction of Germination of Aspergillus Niger Conidia. Fungal Biol..

[38] Bircher L., Sourabié A.M., Paurevic M., Hochuli J., Geirnaert A., Navas C., Drogue B., Lacroix C. (2024). *Faecalibacterium duncaniae* A2-165 Growth Is Strongly Promoted by Yeast Extract and Vitamin B5 in CGMP Medium. Microb. Biotechnol..

[39] Kligler I.J. (1919). Yeast autolysate as a culture medium for bacteria. J. Bacteriol..

[40] Nancib N., Branlant C., Boudrant J. (1991). Metabolic Roles of Peptone and Yeast Extract for the Culture of a Recombinant Strain Of *Escherichia coli*. J. Ind. Microbiol..

[41] Germec M., Turhan I. (2020). Enhanced Production of Aspergillus Niger Inulinase from Sugar Beet Molasses and Its Kinetic Modeling. Biotechnol. Lett..

[42] Waring B.G., Averill C., Hawkes C.V. (2013). Differences in Fungal and Bacterial Physiology Alter Soil Carbon and Nitrogen Cycling: Insights from Meta-analysis and Theoretical Models. Ecol. Lett..

[43] Araújo C.A., Ferreira P.C., Pupin B., Dias L.P., Avalos J., Edwards J., Hallsworth J.E., Rangel D.E.N. (2020). Osmotolerance as a Determinant of Microbial Ecology: A Study of Phylogenetically Diverse Fungi. Fungal Biol..

[44] Stratford M., Steels H., Novodvorska M., Archer D.B., Avery S.V. (2019). Extreme Osmotolerance and Halotolerance in Food-Relevant Yeasts and the Role of Glycerol-Dependent Cell Individuality. Front. Microbiol..

[45] Bubnová M., Zemančíková J., Sychrová H. (2014). Osmotolerant Yeast Species Differ in Basic Physiological Parameters and in Tolerance of Non-osmotic Stresses. Yeast.

[46] Liu G., Chen Y., Færgeman N.J., Nielsen J. (2017). Elimination of the Last Reactions in Ergosterol Biosynthesis Alters the Resistance of Saccharomyces Cerevisiae to Multiple Stresses. FEMS Yeast Res..

[47] Meyer V., Damveld R.A., Arentshorst M., Stahl U., van den Hondel C.A., Ram A.F. (2007). Survival in the Presence of Antifungals. J. Biol. Chem..

[48] Perczyk P., Wójcik A., Wydro P., Broniatowski M. (2020). The Role of Phospholipid Composition and Ergosterol Presence in the Adaptation of Fungal Membranes to Harsh Environmental Conditions–Membrane Modeling Study. Biochim. Biophys. Acta.

[49] Jordá T., Puig S. (2020). Regulation of Ergosterol Biosynthesis in Saccharomyces Cerevisiae. Genes.

[50] Han Z., Zong Y., Zhang X., Gong D., Wang B., Prusky D., Sionov E., Xue H., Bi Y. (2023). Erg4 Is Involved in Ergosterol Biosynthesis, Conidiation and Stress Response in *Penicillium expansum*. J. Fungi.

[51] Turner M.S., Xiang Y., Liang Z.-X., Marcellin E., Pham H.T. (2023). Cyclic-Di-AMP Signalling in Lactic Acid Bacteria. FEMS Microbiol. Rev..

[52] Le Marrec C. (2011). Responses of Lactic Acid Bacteria to Osmotic Stress. Stress Responses of Lactic Acid Bacteria.

[53] Duveau F., Cordier C., Chiron L., LeBec M., Pouzet S., Séguin J., Llamosi A., Sorre B., Di Meglio J.-M., Hersen P. (2023). Yeast Cell Responses and Survival during Periodic Osmotic Stress Are Controlled by Glucose Availability. Elife.

[54] Galello F., Bermúdez-Moretti M., Ortolá Martínez M.C., Rossi S., Portela P. (2024). The CAMP-PKA Signalling Crosstalks with CWI and HOG-MAPK Pathways in Yeast Cell Response to Osmotic and Thermal Stress. Microb. Cell.

[55] Duran R., Cary J.W., Calvo A.M. (2010). Role of the Osmotic Stress Regulatory Pathway in Morphogenesis and Secondary Metabolism in Filamentous Fungi. Toxins.

[56] Zhang Y., Zhu M., Wang H., Yu G., Guo A., Ren W., Li B., Liu N. (2024). The Mitogen-Activated Protein Kinase Hog1 Regulates Fungal Development, Pathogenicity, and Stress Response in *Botryosphaeria dothidea*. Phytopathology.

[57] Wang Y., Liu F., Pei J., Yan H., Wang Y. (2023). The AwHog1 Transcription Factor Influences the Osmotic Stress Response, Mycelium Growth, OTA Production, and Pathogenicity in *Aspergillus westerdijkiae* Fc-1. Toxins.

